# Stigmatizing Monkeypox and COVID-19: A Comparative Framing Study of *The Washington Post*’s Online News

**DOI:** 10.3390/ijerph20043347

**Published:** 2023-02-14

**Authors:** Weilun Ju, Shahrul Nazmi Sannusi, Emma Mohamad

**Affiliations:** 1 Centre for Research in Media and Communication, Faculty of Social Sciences and Humanities, Universiti Kebangsaan Malaysia, Bangi 43600, Malaysia; 2 UKM × UNICEF Communication for Development Centre in Health, Faculty of Social Sciences and Humanities, Universiti Kebangsaan Malaysia, Bangi 43600, Malaysia

**Keywords:** stigma, news frame, monkeypox, COVID-19, *The Washington Post*, online news

## Abstract

Background: Stigma relating to health can result in a broad range of vulnerabilities and risks for patients and healthcare providers. The media play a role in people’s understanding of health, and stigma is socially constructed through many communication channels, including media framing. Recent health issues affected by stigma include monkeypox and COVID-19. Objectives: This research aimed to examine how *The Washington Post* (*WP*) framed the stigma around monkeypox and COVID-19. Guided by framing theory and stigma theory, online news coverage of monkeypox and COVID-19 was analyzed to understand the construction of social stigma through media frames. Methods: This research used qualitative content analysis to compare news framings in *The Washington Post*’s online news coverage of monkeypox and COVID-19. Results: Using endemic, reassurance, and sexual-transmission frames, *The Washington Post* predominantly defined Africa as the source of monkeypox outbreaks, indirectly labeled gays as a specific group more likely to be infected with monkeypox, and emphasized that there was no need to worry about the spread of the monkeypox virus. In its COVID-19 coverage, *The Washington Post* adopted endemic and panic frames to describe China as the source of the coronavirus and to construct an image of panic regarding the spread of the virus. Conclusions: These stigma discourses are essentially manifestations of racism, xenophobia, and sexism in public health issues. This research confirms that the media reinforces the stigma phenomenon in relation to health through framing and provides suggestions for the media to mitigate this issue from a framing perspective.

## 1. Introduction

The COVID-19 pandemic has resulted in enormous challenges and great uncertainty in relation to the health and safety of all human beings [1,2,3]. Similarly, the monkeypox outbreak spread worldwide and threatened global public health security [4,5,6]. The emergence of the monkeypox outbreak—before the world had fully controlled COVID-19 [7]—placed additional burdens on public health systems. Monkeypox, a viral zoonosis caused by the monkeypox virus [8,9], was initially discovered in monkeys in a Danish laboratory in 1958 [10]. The World Health Organization [11] officially declared the monkeypox outbreak a public health emergency in July 2022 [12]. As of 3 October 2022, the WHO [13] reported more than 68,000 confirmed cases of monkeypox globally. Monkeypox has become another significant public health issue in the post-pandemic era.

Monkeypox disease was initially considered endemic to African countries [4,6,9,14], as it had previously spread primarily in sub-Saharan regions [5,8,15]. However, recent outbreaks of monkeypox have occurred more frequently in non-endemic countries [6]. As severe as COVID-19, the United States has become one of the countries most affected by monkeypox outbreaks [13]. As of September 2022, the Centers for Disease Control and Prevention (CDC) [16], an administrative agency of the United States federal government, reported 26,000 confirmed cases, attracting considerable attention from the American media. Media coverage plays a role in framing the monkeypox outbreak that can result in the construction of social stigma around the disease [17,18,19].

Historical experience has shown that certain stigma narratives always emerge in the initial stages of outbreaks [17,20,21,22,23,24]. Stigma is often associated with the use of specific media framing strategies [25,26,27,28,29,30,31,32]. In the era of the COVID-19 pandemic, prejudice against Chinese and Asian people has proliferated in American society [1,23,28,33,34] as online media have favored biased news frames to label China as the predominant “source” of the coronavirus. This has cultivated discriminatory narratives about Asians [35,36,37]. In the early stages of this monkeypox outbreak, African countries were considered the “source” of the spread of the monkeypox virus [18,38,39], a misconception largely produced by media frames. In addition to racism, discrimination around monkeypox disease is also directed at gays [18,19,40], as the initial confirmed cases were incompletely attributed to intimate sex between men [41]. However, there are significant research gaps on the framing of monkeypox issues. Comparing differences in the framing and stigma related to monkeypox and COVID-19 issues will enhance understanding of stigma construction in health, particularly in the post-pandemic era.

In this research, we conducted a qualitative content analysis of *The Washington Post*’s online coverage of monkeypox and COVID-19, guided by news framing theory and stigma theory, to examine and compare how the *WP* frames the stigma surrounding these two diseases.

### 1.1. News Framing Theory

A news frame refers to a mode of selection, emphasis, and exclusion by which the media organize news discourse and construct social meaning [42]. Through news frames, the media remove some elements of perceived facts and reconstruct specific meanings to manipulate reality [43]. When examining the internal devices and construction strategies of the frame, Gamson and Lasch [44] proposed the concept of the interpretive package, which constructs specific symbolic meanings and assigns them to strips of events. It is composed of core frames and positions. The core frame is synthesized as a whole by framing devices (i.e., metaphors, exemplars, catchphrases, depictions, and visual images), while the positions are divided into different parts by reasoning devices (i.e., roots, consequences, and appeals to principles) [44]. Among the framing devices, metaphors refer to rhetorical devices that include a principal subject and an associated subject [44]. Exemplars refer to past or present real events [44]. Depictions refer to the characterization of principal subjects in a specific way, while catchphrases refer to slogans summarizing elaborations regarding principal subjects [44]. Visual images refer to icons or images used to suggest frames [44]. In contrast, among the reasoning devices, roots refer to potential causes, consequences refer to results of specific events, and appeals to principles refer to moral claims or general precepts about events [44].

By analyzing the symbolic elements in the interpretive package, it is possible to elaborate on how a particular news outlet organizes and constructs discourse and meaning around different issues.

### 1.2. Stigma Theory

Goffman [45] initially proposed the concept of stigma, referring to it as an attribute that degrades people from ordinary individuals to “a tainted, discounted one” (p. 3). Labeling theory, proposed by Becker [46], views stigma as a labeling phenomenon whereby something is considered deviant because of specific attributes [47], aptly explaining the underlying mechanisms and salient manifestations of stigma from a semiotic perspective. According to labeling theory, stigma is essentially a labeling or marking behavior that associates particular individuals or groups with stereotypes about them [48]. Negative stereotypes and labels are important components of the stigma. In addition, devaluing stigmatized groups’ social identities [49] is also a component of stigma. Subsequently, stigma is conceptualized systematically [50] and includes labeling differences in specific groups, associating specific groups with their negative stereotypes, separating “us” from “them” (p. 370), making the specific groups lose status, and discriminating against them, as well as reliance on social and political power [50].

Stigma is also defined as social exclusion. Groups with specific attributes are excluded from social interaction due to the stigma around them [51]. Based on this, stigma is regarded as an adaptive behavior in which discrimination against specific groups with perceived threatening attributes is used to protect oneself from uncertain consequences [47]. In other words, stigma is constructed through the exclusion of specific groups perceived to have certain dangerous attributes in order to protect the main group itself from perceived threats. Social exclusion emphasizes the superiority of the self-group [47] and the “otherness” of the marginalized group [19,50].

Naturally, stigmatized groups suffer from various consequences [50]. The immediate consequences of stigma for social outgroups include fear [23,52], abuse and violence [31], social exclusion [51], loss of status, and discrimination [50]. Moreover, stigmatized groups are perceived in terms of “otherness” and marginalization, widening the social divide from the mainstream group [19] and causing stigmatized groups to selectively disclose disease [53] or even voluntarily relinquish power, which is an indirect consequence of stigma.

Utilizing existing theory and research, the mechanism underlying the formation of stigma can be outlined. Negative stereotypes are associated with specific groups with perceived threat attributes when a group perceives uncertain threats [47,48], resulting in devaluation of the specific group’s social identity [45,49], loss of status, discrimination [50], and exclusion [51]. Consequently, stigma is socially constructed.

### 1.3. Stigma around COVID-19 and Monkeypox

Many social epidemiologists regard stigma as an independent social determinant of health. Historical experience shows that stigma is socially constructed [50,54] and closely associated with infectious diseases: from AIDS [21], SARS [22], MERS [20], and Ebola [24] to the current COVID-19 [23,36,53,55] and monkeypox outbreaks [19]. Patients, suspected infected people, or specific groups with attributes associated with infectious diseases are prime targets for stigma [56]. Infectious diseases, especially the COVID-19 pandemic with its high level of uncertainty [2], are more likely to induce stigma around and accusations against social outgroups among “mainstream” groups, allowing them to overcome their psychological anxieties [57,58]. Racism [37,47], xenophobia [52,56], and sexism [59] are the logical underpinnings and cultural norms that make it possible to socially stigmatize and marginalize groups with specific attributes associated with infectious diseases.

With the outbreak of COVID-19, stigmatization of and discrimination against Chinese and Asian people based on racism and xenophobia were rampant [23,34,56] and even led to large numbers of crimes against Asians [1,28,37]. The spread of misinformation was dangerous during the COVID-19 crisis [60]. Misinformation about COVID-19 was widespread in online media [61,62], which exacerbated the stigma surrounding COVID-19. The Trump administration even dubbed the novel coronavirus the “China virus”, paving the way for political blame to be placed on China [33]. Being identified as Asian can significantly increase the likelihood of stigma and discrimination [34].

With the monkeypox outbreak, discrimination and prejudice against the higher-risk groups emerged while the current stigma surrounding COVID-19 was still lingering. First, monkeypox was unilaterally labeled as “endemic to Africa” [63], as it had been largely concentrated on the African continent in the past [5,15]. Stigmatization of African countries may satisfy the colonial mentality of domination and exploitation among the “stigmatizers” [18]. Additionally, stigmatization of gay and bisexual groups was observed, as media reports of confirmed cases were mostly concentrated among men who had sex with men [17], suggesting that the monkeypox outbreaks were closely linked to gay men having sex with men [19]. The sexuality of gay and bisexual people was defined as immoral and negative based on prevailing cultural norms and social traditions [19].

In the COVID-19 pandemic and monkeypox outbreak, stigma has inevitably harmed health and safety and resulted in other sociopolitical consequences.

### 1.4. Stigma and News Frames

Media framing plays a significant role in the construction of stigma. Stereotyping and labeling are prominent features of stigma [45,48]. For instance, news media always use frames to remove elements of perceived realities, reorganize narratives, and construct specific discourses [43] or help construct an identity for the “other” [64]. News frames convey the discourses regarding specific groups and reconstruct their identities (stereotypes), separating “us” (the mainstream group) from “them” (the specific group) [50]. Furthermore, through the symbolic construction of a specific frame, the stereotypes against specific groups with specific attributes are shaped and deepened, making specific groups experience status loss and discrimination [50]. Therefore, the media construct a specific identity (stereotype) for individuals or groups through a specific news frame to socially devalue them [57], and the stigma around individuals or groups is socially constructed and diffused.

The context constructed by media frames provides a breeding ground for stigma and escalates negative stereotypes of stigmatized groups [29]. Labeling is a symbolic interaction process, and the news frame is a symbolic device that media organizations use to construct social meaning [44]. Therefore, media framing is essentially a communication practice that uses specific symbols to label a series of events and groups. Furthermore, news frames with implied stigma in the media may further contribute to the public stigmatization of certain groups [26].

This research identified the following research gaps by integrating empirical research on social stigma and news frames. First, there are significant research gaps relating to the analysis of media frames regarding the monkeypox issue. What remains unknown is how the media use news frames to construct monkeypox stigma. Second, there are apparent research gaps relating to the comparison of media frames and the construction of stigma regarding monkeypox and COVID-19 issues.

Accordingly, we selected *The Washington Post*’s online news coverage of monkeypox and COVID-19 as a case study and investigated how social stigma is constructed. We posed the following research questions:

RQ 1: What news frames are used for monkeypox and COVID-19 in *The Washington Post*?

RQ 2: How does *The Washington Post* frame the stigma regarding monkeypox and COVID-19 issues?

## 2. Materials and Methods

### 2.1. Research Methods

This study used qualitative content analysis to compare the news framing and social stigma regarding monkeypox and COVID-19 issues. Qualitative content analysis involves both manifest and latent content analysis [65,66]. The core objective of this research was to explain the underlying meaning constructed by the news frame; that is, the analysis of latent content. Therefore, qualitative content analysis was judged to be suitable for this study to thematically justify the underlying meanings constructed by a particular frame and its latent connection to the social context.

This study used a qualitative analysis framework to identify latent meanings in frames based on the concept of interpretive packages proposed by Gamson and Lasch [44].

### 2.2. Samples and Data

*The Washington Post*, founded in 1877, is considered a leading elite newspaper in the United States [67,68], with strong influence and news quality [69]. Compared to national newspapers, such as *The New York Times*, the *WP* targets a broader audience [70]. Regarding the selection of the time frame, many studies have confirmed that the early stages of infectious diseases are often accompanied by stigmas and biased narratives targeting specific groups [23,25,29,31,35]. Therefore, news reports from the early monkeypox and early COVID-19 periods were selected to better examine stigma in media coverage. This study defined an “early” time frame as within one month of the day when the first confirmed case was publicly reported. Chinese officials officially reported their first confirmed case of COVID-19 on 31 December 2019 [71], while UK officials reported their first confirmed case of monkeypox on 7 May 2022 [72]. Therefore, the time frame for the COVID-19 reporting sample was 31 December 2019 to 30 January 2020, and the time frame for the monkeypox reporting sample was 7 May 2022 to 6 June 2022.

Online news coverage of monkeypox and COVID-19 was obtained from *WP*’s official website (washingtonpost.com) via keyword searches with the terms “monkeypox”, “coronavirus”, and “COVID-19”. News reports that did not fit the topic were excluded. In the end, 71 online news reports were sampled (N = 71), including 15 monkeypox reports and 56 COVID-19 reports.

### 2.3. Study Instrument

The main coding category involved news frames. This study preliminarily identified five news frames and their operational definitions in existing theoretical and empirical studies [73,74]: the reassurance, attribution of responsibility, governance, panic, and science frames. We also introduced two new frames not defined by existing theories or studies: the endemic frame and the sexual transmission frame. The endemic frame communicates that the health risk is a typical endemic disease within a country or region. The sexual transmission frame communicates that the health risk is caused by a specific sexual activity. A codebook containing complete coding categories and operational definitions was produced and is made available as a Supplementary File.

An inter-coder reliability test was performed with the two coders. The kappa value of the monkeypox sample was 0.814 and that of the COVID-19 sample was 0.817. The percentage of agreement for both reached 80%, as shown in Table 1.

### 2.4. Data Analysis

Analytical methods based on qualitative paradigms were identified. The interpretive package consists of a central frame and positions [44]. Framing devices refer to the symbolic elements of meaning construction, including metaphors, exemplars, catchphrases, depictions, and visual images [44]. Reasoning devices involve roots, consequences, and appeals to principles [44]. The specific positions included within each frame package are listed separately. It should be noted that a frame package does not contain all the devices. Only the devices specifically identified in each frame need to be listed. Through the interaction of specific devices, the positions in the frame package can be determined, which can help explain the underlying meaning of the news frame construction.

After completing the qualitative data analysis of all the samples, we identified the differential discourses constructed by the specific frames according to the patterns of the theme.

## 3. Results

Based on the interpretive package analysis method, the news frames for monkeypox and COVID-19 in the *WP* and the symbolic devices in the frames were identified, as shown in Table 2. Depending on the differential meanings constructed by a particular frame, it was necessary to compare the framing strategies and discourses involved to thematically elaborate on the social stigma around COVID-19 and monkeypox issues.

### 3.1. From “China as the Source” to “Africa as the Source”

The results showed that the *WP* used the same news frame (the endemic frame) to construct different origins for the epidemic. Specifically, the *WP* emphasized that China was the source of the COVID-19 pandemic and that Africa was the source of the monkeypox outbreaks. Although the endemic frame remained unchanged, the differences were apparent in the symbolic devices within the frame and the discourse it constructed regarding COVID-19 and monkeypox issues, as shown in Table 2.

#### 3.1.1. “China as the Source”

With regard to COVID-19 issues, the endemic frame constructed the dominant stigma discourse of “China as the source of the epidemic”. Specifically, the first position directly labeled the coronavirus a “Chinese virus”. The catchphrase “potentially deadly Chinese coronavirus” (*The Washington Post*, 22 January 2020) referred to the virus as the “Chinese coronavirus”, stigmatizing China and arousing more hatred toward China. In the first example below, Chinese people wearing masks were depicted as an iconic image of COVID-19, implying that only Chinese people wear masks because of the coronavirus and conveying negative stereotypes about China.

*Pedestrians in disposable face masks have become a defining image of the coronavirus outbreak in China (The Washington Post, 25 January 2020).*

The second position defined Wuhan as the source and severely stigmatized the citizens of Wuhan. The *WP* used the metaphor “Specter of possible new virus emerging from central China” (*The Washington Post*, 8 January 2020) to define China as the source of the coronavirus. The catchphrase “Wuhan: The Chinese mega-city at the center of coronavirus outbreak” (*The Washington Post*, 23 January 2020) directly described Wuhan as the source of the outbreak. In addition, the frame also used the following reasoning devices to consolidate this position. In the second example, the root, a reasoning device, was used to directly define Wuhan as the source of the epidemic and to label the citizens of Wuhan as “virus carriers”, which stigmatized the Chinese people, especially the citizens of Wuhan, and could have led to instigating hatred toward them.

*Cases in China continue to multiply, and five million residents of Wuhan, where the virus originated, have left the city, some of them surely carrying the disease (The Washington Post, 29 January 2020).*

**Table 2:** Framing analysis of monkeypox and COVID-19 issues.

Issue	News Frame	Positions	Framing Devices					Reasoning Devices		
			Metaphors	Exemplars	Catchphrases	Depictions ^a^	Visual Images	Roots	Consequences	Appeals to Principles
**COVID-19**	Endemic	“China’s coronavirus” or “Chinese coronavirus”			An article mentioned the first US case of infection with the potentially deadly “Chinese coronavirus”	“Pedestrians in disposable face masks have become a defining image of the coronavirus outbreak in China”				
		Citizens leaving Wuhan, the source of the outbreak, carried the virus	An article mentioned that the specter of COVID-19 emerged from central China		An article mentioned that Wuhan was at the center of the coronavirus outbreak			An article mentioned that residents carrying the disease left Wuhan, where the virus originated		
	Panic	Mysterious and scary coronavirus				“What we know about the mysterious, pneumonia-like coronavirus spreading in China and elsewhere”				
		The healthcare system was in trouble				“There are not enough hospitals and not enough beds, not enough doctors and not enough nurses, not enough rubber gloves and not enough face masks”				
		The epidemic caused widespread worries among the public		An article mentioned individual stories of people freaking out, snapping up masks, and even leaving Wuhan in a hurry		“Some early signs are discouraging: Six countries, including China, have confirmed human-to-human transmission of the infection”			An article mentioned that several Tokyo Olympic 2020 qualifying events were rescheduled	
	Attribution of responsibility	The Chinese government was responsible for the untimely or inadequate reporting of the epidemic		An article mentioned that local authorities were slow to report the new disease, as they were with SARS at the time	An article mentioned that China should learn lessons from SARS	“Nor have Chinese officials provided a timeline of patient illnesses, information that is typically made public quickly in disease outbreaks”				
		China has previously failed to effectively control the illegal wildlife trade		An article quotes an expert as saying that China was suffering the costs of not learning the lessons of SARS and controlling the illegal wildlife trade		“But the regulations will remain in place only while China grapples with the epidemic, raising the question of whether the wildlife trade will be allowed to bounce back, as it did after an initial clampdown following the SARS outbreak”	A picture shows that Chinese police investigated an illegal site for wildlife trafficking	An article mentioned that the virus originated in Wuhan’s South China Seafood City, which trades in wild meat		
	Governance	Various measures have been taken to deal with COVID-19							An article mentioned that North Korea banned foreign tourists. Flights between Wuhan, China, and the US were banned	
	Science	The novel coronavirus is the cause of COVID-19						An article mentioned that Chinese researchers isolated the coronavirus, a cause of COVID-19		An article mentioned that the risks of xenophobia for human rights should bring wide concern
**Monkeypox**	Endemic	Monkeypox has usually occurred in Africa, so it was unusual and rare to find many cases in Europe and America			An article mentioned that the case of monkeypox confirmed in Massachusetts was rare	“Experts say the latest cases are unusual because of the level of spread among patients with no known travel history to Africa”		An article mentioned that monkeypox spread among “non-endemic” countries		
		It was necessary to investigate the African travel history of patients and to refrain from consuming African wildlife products				“‘None of these people reported having recently been in central or west African countries where monkeypox usually occurs…’”			An article mentioned that travelers were banned from buying products made from African wildlife	
	Reassurance	The vaccines were sufficient and effective, so there was no need to worry as there was with COVID-19	An article mentioned that monkeypox was not the next coronavirus pandemic	An article mentioned that the smallpox vaccine was 85% effective against monkeypox	An article mentioned that there was no need to panic about monkeypox	“President Biden said… there are sufficient vaccine doses available to combat any serious flare-up of the disease” “And monkeypox has key differences from the fast-moving, more-transmissible coronavirus...”				
	Sexual transmission	Men who had sex with men were more likely to be infected with monkeypox			An article mentioned that monkeypox patients should abstain from sex	“… most confirmed cases have been identified in gay, bisexual and other men who have sex with men, and that the risk to the wider population is ‘low’”				

Note: ^a^ The expressions in the Depictions column are all taken from news articles in *The Washington Post*.

#### 3.1.2. “Africa as the Source”

In another discourse, African countries have been stigmatized as “the original source” of monkeypox outbreaks. There are two main positions in this discourse. The first position reinforced an old stereotype that “monkeypox is an endemic disease in African countries”, so it was rare and unusual to find cases in Europe and the United States. The catchphrase “Rare monkeypox case confirmed in Massachusetts” (*The Washington Post*, 19 May 2022) emphasized that monkeypox was a rare phenomenon in the United States, implying that confirmed cases in Africa were common. Similarly, the first example below quoted an anonymous expert, where the word “unusual” reinforces the position that cases in the United States are rare. In other words, only confirmed patients with a history of travel to Africa are normal, and this discourse undoubtedly implies that Africa is the source of monkeypox. However, there is currently no evidence that confirmed cases in European and American countries are related to travel to Africa [17,75], so the depiction is biased. Moreover, the reasoning device of the root was used to define Africa as the typical source of monkeypox outbreaks.

*Experts say the latest cases are unusual because of the level of spread among patients with no known travel history to Africa (The Washington Post, 21 May 2022).*

The second position claimed that it was necessary to investigate the travel history of patients in Africa and to refrain from buying African wildlife products. In the second example, the depiction of monkeypox patients being investigated for travel history in Africa reinforced the discourse that Africa was inextricably linked to the outbreak of monkeypox and reaffirmed the “Africa is the source” label. In the third example, the ban on African wildlife products was highlighted as a consequence of the monkeypox epidemic, which resulted in an invisible social distancing of African countries. The second and third examples are shown below:

*“None of these people reported having recently been in central or west African countries where monkeypox usually occurs, including the Democratic Republic of the Congo and Nigeria, among others,” the CDC alert says (The Washington Post, 26 May 2022).*

*… travelers should refrain from using products… made from African wild animals (The Washington Post, 26 May 2022).*

Thus, despite using the same endemic frame for COVID-19 and monkeypox, the *WP* constructed differentiated social stigmas and epidemic discourses based on differences in internal symbolic devices. From the COVID-19 pandemic to the monkeypox epidemic, the objects and groups at which the stigma was directed changed qualitatively—from China to African countries. Both China and Africa were symbolically labeled “the source of the epidemic” and, moreover, the citizens of Wuhan were directly labeled as “virus carriers”, which may have caused the corresponding groups to suffer from varying degrees of social distancing and racial discrimination.

### 3.2. From Panic to Reassurance

For COVID-19 and monkeypox, the *WP* used the panic and reassurance frames, respectively, to construct public perceptions and attitudes toward different outbreaks, emphasizing that COVID-19 was worrying or frightening while monkeypox was not something to be overly worried about. The panic and reassurance frames are a pair of competing frames in terms of discourses and meanings, facilitating the *WP*’s construction of differential discourses for COVID-19 and monkeypox outbreaks, as shown in Table 2.

#### 3.2.1. Panic and COVID-19

Panic frames were used to construct discourses about the coronavirus scare in response to the COVID-19 issue. Specifically, there were three main positions. The first position described the novel coronavirus as a mysterious and terrifying virus. For example, the depiction “What we know about the mysterious, pneumonia-like coronavirus spreading in China and elsewhere” (*The Washington Post*, 22 January 2020) created an atmosphere of high uncertainty. The second position claimed that China’s healthcare system had encountered obstacles as the outbreak intensified. For instance, the first example below described the shortage of medical supplies and personnel in China, emphasizing that China’s health system was overwhelmed and constructing a discourse of panic.

*There are not enough hospitals and not enough beds, not enough doctors and not enough nurses, not enough rubber gloves and not enough face masks (The Washington Post, 25 January 2020).*

Based on the foreshadowing of the first two positions, the third position, “The spread of the epidemic has caused widespread public worries”, logically became the mainstream position of the panic frame, in which various exemplars based on individual stories and feelings were used. For example, in the second example, the personal experiences of foreigners living in China were invoked through exemplars, a framing device, to claim that the locals were worried and rushing for masks. Similarly, the third example again used exemplars to convey the anxiety of a local citizen who wanted to leave Wuhan. These exemplars subtly applied the label “out of control and chaos in the early COVID-19” to China—for instance, by discussing masks that quickly sold out and citizens being eager to leave Wuhan—greatly stimulating the audience’s anxiety, worries, and fear about the coronavirus. In addition, the fourth example described the signs of human-to-human transmission as “discouraging”, which increased the fear of China. Finally, the frame proposed consequences caused by COVID-19 concerns—several Tokyo 2020 Olympic qualifying events were rescheduled, highlighting global concerns about the epidemic. The second, third, and fourth examples are as follows:

*Though his girlfriend (* *“his girlfriend” refers to an ordinary Korean-American studying in China) lives far from Wuhan, everyone around her is freaking out, she’s told him. Whenever someone offers surgical masks for sale online, she said, the masks are gone within minutes (The Washington Post, 27 January 2020).*

*“Whatever train ticket I can get, as long as I can get out of Wuhan,” one would-be passenger at Han-kou station told a local reporter early Thursday (The Washington Post, 22 January 2020).*

*Some early signs are discouraging: Six countries, including China, have confirmed human-to-human transmission of the infection (The Washington Post, 29 January 2020).*

#### 3.2.2. Reassurance and Monkeypox

In contrast to the discourse conveyed in the COVID-19 coverage, the *WP* mainly used the reassurance frame for monkeypox issues and constructed the news discourse that the outbreak was nothing to worry about. The position of the frame claimed that the US vaccine was sufficient and effective and that there was no need to worry as there had been with COVID-19.

First, the metaphor “Monkeypox isn’t the next coronavirus” and catchphrase “No need to panic about monkeypox” (*The Washington Post*, 24 May 2022) were utilized to separate monkeypox from COVID-19 and to reassure the audience that the monkeypox outbreak was not a new round of the pandemic. The first example below used the exemplar “high efficacy of smallpox vaccine” to prove that, even if there was no unique vaccine against the monkeypox virus, the alternative vaccine—the smallpox vaccine—was still effective in preventing monkeypox.

*Studies suggest that the smallpox vaccine is at least 85 percent effective against monkeypox… (The Washington Post, 22 May 2022).*

In addition, the *WP* used several depictions to further “cool down” the severity of the monkeypox. For example, the second example paraphrased President Biden’s views to appease anxiety and increase public confidence in the government. The third example emphasized that the danger and transmissibility of monkeypox differed from those of the coronavirus, suggesting that the public should not be overly worried about monkeypox outbreaks. The second and third examples are shown below:

*President Biden said… there are sufficient vaccine doses available to combat any serious flare-up of the disease (The Washington Post, 23 May 2022).*

*And monkeypox has key differences from the fast-moving, more-transmissible coronavirus that make it easier to contain and treat (The Washington Post, 24 May 2022).*

Thus, the *WP* used the panic and reassurance frames for COVID-19 and monkeypox outbreaks, respectively, to construct opposing news discourses. First, labeling China as “panicky and disorderly” have bred public distrust and fear of China. In contrast, the reassurance frame repeatedly emphasized the sufficient and effective vaccine stockpiles in the United States to soothe the emotions of the American people and increase public confidence in the federal government’s vaccine policy. In short, the *WP* adopted a reassurance discourse in the face of the spread of the monkeypox epidemic in the West, while defining the spread of the coronavirus in China as a dire threat.

### 3.3. From Blaming the Government to Labeling Gays

In addition, as shown in Table 2, the attribution of responsibility frame was used to blame the Chinese government for inadequacies during the COVID-19 crisis, while the sexual transmission frame indirectly labeled gays as a specific group more likely to be infected with monkeypox.

#### 3.3.1. Blaming the Government

The *WP* constructed a discourse blaming the Chinese government through the attribution of responsibility frame. There were two main positions. The first position, derived from the labels “Wuhan as the epicenter” and “China as the source”, claimed that the Chinese government, especially the local government in Wuhan, was responsible for the spread of the epidemic. First, the *WP* cited the exemplar that the Wuhan local government was slow to report new cases, as it had been during the previous SARS period. Concurrently, the *WP* also used the catchphrase that China should learn from previous outbreaks (i.e., SARS), claiming that China had not learned lessons from SARS and blaming the local government in Wuhan for not releasing epidemic data promptly. The first example below claimed that the government’s disclosures were inadequate, blaming the government for not releasing more information about the outbreak as expected.

*But while Chinese officials have ruled out several causes of the illnesses, they have not provided detailed information about what tests were performed, when they were done and at what point in the patients’ illnesses. Nor have Chinese officials provided a timeline of patient illnesses, information that is typically made public quickly in disease outbreaks (The Washington Post, 8 January 2020).*

The second position claimed that China had previously failed to effectively control the illegal wildlife trade. This position defined “Wuhan’s South China Seafood City” as the root of all problems, emphasizing that it was precisely because the Chinese government had not properly controlled the wildlife trade that the epidemic had broken out in Wuhan’s seafood market. Moreover, a visual image showed Chinese police investigating an illegal wildlife trade site. On the surface, the image communicated that the government was taking action and cracking down on the illegal wildlife trade. In fact, however, it emphasized that such illegal trade did exist in China and could easily lead to the spread of infectious diseases. This framing strategy subtly blamed the government for its mistakes, stigmatized China, and deepened public hatred and discrimination against Chinese people. The first example below quoted an anonymous expert who claimed that the spread of the coronavirus was a consequence of the Chinese government’s failure to learn from SARS and ban the wildlife trade. The second example again used SARS as an exemplar to blame the government for being responsible for the proliferation of the wildlife trade and to raise suspicions that the trade-ban policy may be just a temporary stopgap measure in the face of the epidemic crisis.

*Experts say the country is paying a heavy price after the government failed to learn one of the most important lessons of the SARS… that diseases can easily mutate and spread to humans in markets where different species of live wild animals are kept in proximity… (The Washington Post, 26 January 2020).*

*But the regulations will remain in place only while China grapples with the epidemic, raising the question of whether the wildlife trade will be allowed to bounce back, as it did after an initial clampdown following the SARS outbreak… Peter Knights, founder of WildAid, said the current crisis might have been averted if the ban after SARS had been permanent (The Washington Post, 26 January 2020).*

*As with the MERS and SARS viruses, the Wuhan pneumonia is caused by a novel coronavirus that jumped from animals to people. Poor animal husbandry in open food markets make China an epicenter for these risks. Wuhan’s bazaar sells freshly slaughtered animals—including chickens, pheasants, marmots, snakes, deer and rabbits. These “wet markets” create perfect conditions for viral species to spread from their animal hosts to humans (The Washington Post, 23 January 2020).*

Additionally, the third example directly defined Wuhan as the source of COVID-19 because of the rampant wildlife trade through depictions of “the Wuhan pneumonia”, “an epicenter”, and “Wuhan’s bazaar”. Moreover, the depiction “As with the MERS and SARS viruses”, while ostensibly emphasizing the similarities between COVID-19 and previous viruses, implied that the origins of this outbreak were also similar to those of previous outbreaks—for both, wildlife trade was not fully banned, and the depiction thus indirectly attributed the outbreak to the government’s failure to permanently ban this trade. In short, these examples not only blamed the Chinese government for the outbreak but also indirectly reinforced the inherent stigma that Wuhan, China, was the source of COVID-19 because of the wildlife trade.

#### 3.3.2. Labeling Gays

This study found that the *WP* used the sexual transmission frame, indirectly labeling gay communities as a group more likely to be infected with monkeypox. The frame inadvertently constructed the position that men who have sex with men were more likely to be infected with monkeypox. First, the catchphrase “Monkeypox patients should abstain from sex while symptomatic” (*The Washington Post*, 31 May 2022) associated monkeypox with sexual behavior, implying that sexual behavior was inextricably linked to the outbreak. In addition, in the following example, the depiction “predominantly in gay… men” not only associated monkeypox with men who have sex with men but also unintentionally labeled gays and other related groups as “the specific population” more likely to be infected with monkeypox.

*British health authorities said Wednesday that nine infections have been confirmed in England since May 6, “with recent cases predominantly in gay, bisexual or men who have sex with men.” (The Washington Post, 19 May 2022)*

Furthermore, the first example below used the depiction “most confirmed cases” to indirectly describe gays and other related groups as people who were more likely to be infected with monkeypox, potentially blaming gays who have sex with men for spreading the monkeypox. Moreover, the term “wider population” in this example invisibly defined gay communities as minority groups and differentiated them from so-called “mainstream” groups. The term “low” was used to claim that the risk of infection in the “wider population” was low, indirectly emphasizing that gays were more likely to be infected with monkeypox than the “wider population”. The consequences of this included not only the marginalization and stigmatization of gays, but may also have caused other groups to relax their vigilance, increasing the risk of virus transmission. Therefore, the terms “wider population” and “low” labeled gays as “a special group more likely to be infected” and facilitated the marginalization and discrimination against them.

*Rosamund Lewis, the WHO’s technical leader on monkeypox, said… most confirmed cases have been identified in gay, bisexual and other men who have sex with men, and that the risk to the wider population is “low” (The Washington Post, 31 May 2022).*

*Federal officials say they are working with gay social networking apps and LGBTQ organizations to spread the word about monkeypox ahead of Pride Month in June. They say there is no reason to avoid festivities, but people should avoid contact with those with rashes and seek medical care if they develop unusual rashes (The Washington Post, 27 May 2022).*

In the second example, officials specifically disseminated knowledge about monkeypox and medical reminders to homosexuals. Although its original intention was to emphasize the importance of disseminating knowledge about monkeypox to homosexuals, it also indirectly constructed a discourse stating that knowledge about monkeypox was to be specifically disseminated to homosexuals because they might be more likely to be infected with monkeypox. Simultaneously, the depiction “… no reason to avoid festivals, but people should avoid contact with those with rashes…” inadvertently implied that people attending festivities might be infected with rashes—a typical clinical manifestation of monkeypox. The dissemination of knowledge and medical reminders specifically targeted at the homosexual group might have made them appear as special cases requiring special attention, indirectly separating them from the “mainstream” group and reinforcing the marginalization against them, especially gays. For example, “gay social networking apps” alluded to the fact that the target audience for spreading knowledge in this context was mainly gay people.

Thus, the attribution of responsibility frame blamed the government’s inadequacies for the early spread of the coronavirus, further reinforcing the stigmatization of the Chinese people. The sexual transmission frame indirectly defined gays as a specific group who were more likely to be infected, thus constantly marginalizing gay people during the monkeypox epidemic. In essence, these frames all constructed social stigmas around a health crisis.

## 4. Discussion

This study conducted a qualitative content analysis guided by news framing theory and stigma theory to examine how *The Washington Post*’s (*WP*) online news framed stigma regarding monkeypox and COVID-19. The results confirmed that there were meaningful differences in the stigmas constructed by the *WP* regarding monkeypox and COVID-19. It predominantly used endemic, reassurance, and sexual transmission frames regarding the early monkeypox epidemic, which differed from the endemic, panic, and attribution of responsibility frames in the COVID-19 coverage. Based on the different frames, the *WP* accordingly constructed differential stigmas for early COVID-19 and the monkeypox outbreaks, moving from stigmatizing China to stigmatizing Africa or indirectly labeling gays as a specific group more likely to be infected with monkeypox and from the coronavirus spreading in China being worthy of panic to the monkeypox spreading in the United States being nothing to worry about. The differentiated stigma discourses are essentially manifestations of racism, xenophobia, and sexism in public health issues.

In particular, the stigmatization of Africa and China is essentially a form of health racism. Despite the shift in stigmatized objects and groups, from COVID-19 to monkeypox, the essence of this framing strategy—racism—remains the same. Stigma theory suggests that, when a group perceives a real threat, it attempts to transfer the perceived threat to the victim group by associating negative stereotypes with the victim group. This labeling strategy results in the compromised [50] and degraded status [45,49] of the victim group, contributing to stigma based on ethnocentrism [47]. Specifically, this study found that the endemic frame labeled African countries as the “typical source of monkeypox” in monkeypox outbreaks, enriching the literature on stigma and contributing to framing studies of monkeypox outbreaks. The *WP* associated African countries with the stereotype of the monkeypox epidemic and emphasized that monkeypox was not the “patent” of the West, which shifted the perceived threat around monkeypox from Western countries to Africa. Thus, stigmatization of African countries followed, as for COVID-19 and China. In response to COVID-19, the endemic frame mainly defined China as the “source of the coronavirus” and used the label “Chinese virus”, a finding similar to those of previous framing research involving COVID-19 stigma [25,29,35,37]. The framing strategy constructed racial stigma around the Chinese by shifting the perceived threat to China. However, when the monkeypox epidemic was first reported in the United Kingdom and spread widely in European and North American countries, the *WP* did not use the labels “British virus” or “American virus”. It simply emphasized that it was a typical disease in Africa and that patients’ African sojourn histories needed to be investigated. Negatively framing certain groups with biased frames is a potential cause exacerbating racism and xenophobia [29,37]. Therefore, shifting perceived threats to Africa or China essentially amounts to constructing racist stigmas, resulting in hatred of and discrimination against Africa and China.

Furthermore, allaying worries about the homegrown monkeypox outbreak and stigmatizing the spread of the coronavirus in China are essentially forms of xenophobia and Western-centrism. Xenophobia refers to the psychology of exclusion arising from fear of or dissatisfaction with outgroups [76] and is inseparable from the psychological fears of the population [52], especially during epidemics, because the public’s fear of infectious diseases is far greater than for other diseases [52]. Based on the relationship between fear and xenophobia, the *WP* used the panic frame in its COVID-19 coverage to render the spread of the coronavirus in China into an image of “chaos and panic”, making xenophobia against Chinese and Asian people rampant [56]. In contrast, due to the outbreak of monkeypox in Europe and the United States, the *WP* preferred the reassurance frame rather than the panic frame to eliminate the anxiety around and fear of monkeypox among the American people and to emphasize that the vaccine in the United States was sufficient and effective. In addition, emphasizing the effectiveness of the vaccine increased the public’s confidence in the US federal government’s health policies. The *WP* adopted a reassuring discourse as the monkeypox epidemic spread in the West. In contrast, the spread of the coronavirus in China was framed as a dire threat, which indicated the American media’s differential framing strategies, which are pro-Western and exclude outgroups, as proposed in previous research [67,77]. Therefore, based on xenophobia and Western-centrism, the *WP* tended to allay worries about the monkeypox epidemic spreading in Europe and the United States and to construct panic about the pandemic in China, which resulted in more Chinese and Asian people being excluded and discriminated against.

A case in point is the statement that “…five million residents of Wuhan, where the virus originated, have left the city, some of them surely carrying the disease” (*The Washington Post*, 29 January 2020). This example not only implied a racist stigmatization of the citizens of Wuhan but also inspired fear of and xenophobia against the Chinese people. To alleviate the inherent stigma, the stigmatizing frame can be corrected into a non-stigmatizing frame by simply adjusting some of the framing devices. In this case, the stigmatizing depictions “where the virus originated” and “carrying the disease” can be removed. Accordingly, the original expression can be revised to “five million residents of Wuhan have left the city”, which is a non-stigmatizing frame. With the removal of the stigmatizing devices, the non-stigmatizing frame no longer defines Wuhan as the source, nor does it breed fear and xenophobia toward Wuhan residents, effectively reducing the stigma surrounding COVID-19. The non-stigmatizing frame restores the objectivity and authenticity that news coverage should have.

Additionally, gays were indirectly labeled as a specific group more likely to be infected with monkeypox, even if some coverage did not directly stigmatize gays, which invisibly marginalized gays. This framing strategy may be inseparable from inherent sexist discrimination. Sexism is a system of inequality based on a gender ideology that maintains dominance [78]. Based on the conventional gender ideology, the sexuality of gay communities is stereotyped as promiscuous and immoral behavior [19]. Stigma is a marking behavior that associates negative stereotypes with specific groups [48]. In order to maintain the mainstream status of gender ideology, homosexual groups are often associated with stereotypes of immoral sexual behavior, resulting in the emergence of sexist stigma regarding infectious disease issues [59].

For example, the *WP* quoted an expert’s statement and emphasized that “… most confirmed cases have been identified in gay, bisexual and other men who have sex with men, and that the risk to the wider population is ‘low’” (*The Washington Post*, 31 May 2022). By associating the sexuality of gay communities with monkeypox, sexual transmission frames indirectly labeled gays who have sex as “a specific group more likely to get monkeypox”, thus separating gay communities from social groups. This framing strategy reinforced the marginalization and exclusion of gays based on conventional gender ideology. Admittedly, the *WP* also used some non-stigmatizing frames in its coverage of monkeypox, such as “…it doesn’t matter what your age or gender or sexual orientation is: Anyone could potentially be exposed to monkeypox…” (*The Washington Post*, 27 May 2022). It avoided differentiating gay people from the social group and emphasized everyone’s equality before the virus. This non-stigmatizing frame not only avoided stigmatizing framing devices but also objectively conveyed the scientific facts, excluded by the sexual transmission frame, that anyone is likely to be infected with monkeypox.

Consequently, the findings of this study have practical implications for how the media report health crises in a neutral manner. Regarding public health crises, how the news media adopt a non-biased framing strategy to alleviate existing stigma is crucial, as the media play an important role in delivering health information during health crises [79]. First, the media can focus on more non-stigmatizing frames rather than stigmatizing ones. On the one hand, the media need to avoid framing devices with stigmas, such as the “Chinese virus”, “African countries where monkeypox usually occurs”, and “predominantly in gay… men”, and convey objective facts and scientific information about the health crisis. In particular, when referring to a specific region or group, the media should not only avoid using disease terms with the name of the region or group but also discard unnecessary modifiers, such as “where monkeypox usually occurs” or “predominantly”. On the other hand, the media can try to add balanced scientific information if a reference to specific regions or groups is unavoidable. For example, when reporting on monkeypox, the media can, while mentioning gays, emphasize that anyone can be infected with the virus, which could effectively balance the public’s special attention toward and marginalization of a particular group. Finally, journalism can focus on cultivating and improving health journalists’ scientific literacy and professional ethics, which could limit the construction of stigma at the source of journalistic production.

Nevertheless, there are limitations to this study. Although the findings of this study indicate that the relevant coverage of the *WP* constructed and reinforced the stigmas surrounding monkeypox and COVID-19, this does not mean that all of its coverage constructs stigmas and or that all media will construct stigmas, as this study focused only on the online coverage of one English-language newspaper. Moreover, this study did not examine potential associations between the disease characteristics of monkeypox or COVID-19 and the differences in the stigmas surrounding monkeypox and COVID-19. Future research could attempt to examine the broader international reporting on monkeypox and COVID-19 issues and decipher the differences and similarities in the stigmas surrounding monkeypox and COVID-19 based on their disease characteristics.

## 5. Conclusions

In conclusion, the news media used biased frames to construct stigmas, which could have contributed to creating a worse “epidemic”. By examining the online coverage of monkeypox and COVID-19 in *The Washington Post*, this study found that it mainly used endemic, reassurance, and sexual transmission frames to construct stigma surrounding monkeypox, which differed from the endemic, panic, and attribution of responsibility frames used for COVID-19 issues. Different stigmas were constructed accordingly for early COVID-19 and the monkeypox outbreaks: the *WP* moved from stigmatizing China to stigmatizing Africa and indirectly labeling gays as a specific group more likely to be infected with monkeypox; and from claiming that the coronavirus spreading in China was worthy of panic to emphasizing that monkeypox spreading in the United States was nothing to worry about. Although the stigmatized groups and the discourses of stigma were different, the nature of the discourses—racist, xenophobic, and sexist—did not change. From COVID-19 to monkeypox, *WP*’s framing construction is essentially a form of racism and sexism in public health. This research provides constructive recommendations for the media to mitigate stigma.

## Figures and Tables

**Table 1:** Kappa statistics and coding agreement for the samples for the monkeypox and COVID-19 issues.

Coding Category	Kappa Value (κ)	Percentage of Agreement	Total Items in Sample
Monkeypox coverage	0.814	87.5%	8
COVID-19 coverage	0.817	84.6%	13

## Data Availability

Data availability upon request to the authors.

## Supplementary Materials

The following supporting information can be downloaded at: https://www.mdpi.com/article/10.3390/ijerph20043347/s1, Table S1: Framing Codebook for Monkeypox and COVD-19 Issues.
