# Supplementary material for: Stigmatizing Monkeypox and COVID-19: A Comparative Framing Study of The Washington Post’s Online News
Source: Int J Environ Res Public Health. 2023 Feb 14;20(4):3347. doi: 10.3390/ijerph20043347 (PMC9965175; doi:10.3390/ijerph20043347)
Supplement: Supplementary file 1 [file ijerph-20-03347-s001.zip › ijerph-2142163-supplementary.pdf]

# Supplementary Materials

## Framing Codebook

**Table S1: Framing Codebook for Monkeypox and COVD-19 Issues**

| News Frame (Coding Value) | Operational Definition | Example |
|---|---|---|
| Endemic (1) | The health risk is a typical endemic disease within a country or region. | Monkeypox is rare in Europe because it was once considered endemic to Africa. |
| Sexual Transmission (2) | The health risk is caused by specific sexuality. | Most of the confirmed cases have been reported in men who have had intimate sex with men. |
| Reassurance (3) | There is no need to worry about health risks as they can be treated. | Sufficient and effective vaccines will be available, so there is no need to worry about it. |
| Panic (4) | The health risk has caused widespread concern and fear. | The rapid spread of COVID-19 has caused widespread concern and fear. |
| Attribution of Responsibility (5) | The health risk is being blamed on specific individuals or groups. | The government has a responsibility to stop the illegal wildlife trade. |
| Governance (6) | Governments take necessary policies or measures to deal with health risks. | The government takes a series of effective lockdown measures to control the spread of the virus. |
| Science (7) | The health risk is objectively explained on a scientific basis. | Researchers measured and compared the viral gene sequences. |
